# The RECLAIM adaptive platform trial for the evaluation of treatments for post-COVID condition in the Netherlands: core protocol

**DOI:** 10.1186/s13063-026-09570-1

**Published:** 2026-02-19

**Authors:** Janneke H. H. M. van de Wijgert, Julie M. H. Schoevers, Todd D. Swarthout, Wietske J. T. Bouwman, Joost van Rosmalen, Evelien R. Leffelaar, Arief Lalmohamed, Chantal Loch, Cees C. van den Wijngaard, Cristina Prat-Aymerich, Joost J. Schotsman, Eveline D. Verheijen, Johanna G. van der Bom, Marc J. M. Bonten

**Affiliations:** 1 Julius Center for Health Sciences and Primary Care, University Medical Center Utrecht, Utrecht University, Universiteitsweg 100, Stratenum Room 6.131, Utrecht, 3584 CG The Netherlands; 2 European Clinical Research Alliance on Infectious Diseases (Ecraid), Utrecht, The Netherlands; 3 Pharmacy, University Medical Center Utrecht, Utrecht University, Utrecht, The Netherlands; 4 Centre for Infectious Disease Control, National Institute for Public Health and the Environment (RIVM), Bilthoven, The Netherlands; 5 Department of Clinical Epidemiology, Leiden University Medical Center, Leiden, The Netherlands

**Keywords:** Post-COVID condition, Adaptive platform trial, Metformin, Colchicine, Minocycline

## Abstract

**Background:**

Post-COVID condition (PCC) encompasses a heterogeneous spectrum of pathological responses to SARS-CoV-2 infection, with unknown individual predispositions. No evidence-based, curative treatments are available. Many patients therefore resort to off-label use of medications originally developed for other indications. However, the efficacy and safety of repurposed drugs in the context of PCC are unknown.

**Methods:**

RECLAIM is a phase III, randomised, controlled, adaptive platform trial. The trial population consists of adult patients who have had persistent PCC symptoms, including fatigue and/or post-exertional malaise, 12 or more weeks after the onset of a SARS-CoV-2 infection. Randomisation occurs within trial domains. A trial domain consists of one or multiple investigational products (IPs), each to be compared to one usual care arm, or one or multiple IPs, each to be compared to a matching placebo. Within each trial domain, patients can be enrolled if they are eligible for at least one IP and its control. They are randomised with equal probability to each arm for which they are eligible. Trial product use (if applicable) starts as soon as possible after randomisation for a default duration of 12 weeks. Study procedures are implemented remotely, using video consultations, eConsenting, trial product delivery to participants’ homes (if applicable), and online questionnaires at baseline, day 1, and weeks 2, 4, 6, 8, 10, 12, and 24. The primary outcome is health-related quality of life at week 12, assessed by Patient-Reported Outcomes Measurement Information System Profile29 (PROMIS-29) physical health summary scores, and is analysed with a Bayesian analysis of covariance model with adjustment for the baseline value. Secondary outcomes include week 12 PROMIS-29 mental health summary scores and topic domain scores; specific PCC symptoms using other patient-reported outcome measures, safety, and tolerability; and durability of treatment responses at week 24. Participants are allowed to participate in only one trial domain at a time, but can be rerandomised into a different trial domain after completing week 24.

**Discussion:**

RECLAIM commenced in February 2025 with an open-label domain including two IP arms (metformin and colchicine) and one usual care control arm. A second placebo-controlled domain, comparing minocycline to a matching placebo, was initiated in February 2026.

**Trial registration:**

European Clinical Trials Information System (CTIS) EUCT number 2024-511580-28-02. Registered on 9 October 2024. ClinicalTrials.gov identifier NCT07280572. Registered on 11 December 2025.

**Supplementary Information:**

The online version contains supplementary material available at 10.1186/s13063-026-09570-1.

## Background

Post-COVID condition (PCC)concerns a heterogeneous syndrome of long-term health problems after an acute SARS-CoV-2 infection. Symptoms are diverse, with general fatigue, post-exertional malaise (PEM), cognitive problems, and postural orthostatic tachycardia syndrome (POTS) being prominent [1, 2]. Attempts have been made to define PCC clinical phenotypes, but these are not (yet) consensus-based [3–5]. PCC can be devastating, impacting significantly on quality of life and social functioning. PCC incidence is very difficult to estimate for several reasons, including differences between SARS-CoV-2 strains in risk of developing PCC and limited national PCC surveillance [6, 7]. There are estimated to be more than 90,000 PCC patients in the Netherlands whose symptoms are severe and have been ongoing for more than 1 year [8]. The causal mechanisms are still unknown but various hypotheses have been postulated, including cell, tissue, and/or organ damage by the SARS-CoV-2 virus itself or by reactivation of latent viruses; dysregulation of the immune system triggered by the infection; and/or obstruction of small blood vessels due to endothelial damage or micro-thrombi [9, 10]. These processes may subsequently dysregulate the central nervous system, e.g. due to neuro-inflammation. There is currently no standard of care for PCC and no agreed upon treatment for most PCC symptoms. Current PCC management is focused on relieving specific symptoms on an individual trial-and-error basis [11, 12].

While the development of new PCC-specific treatments will likely take many years, existing medications already approved for other conditions (repurposed drugs) can be evaluated in the meantime for their potential to alleviate symptoms and enhance the quality of life in PCC patients. In fact, many PCC patients and their physicians are already trying repurposed drugs, even though their efficacy and safety in PCC patients have not been formally evaluated. Conducting multiple randomised clinical trials in parallel or in sequence is time- and resource-intensive, even when evaluating repurposed drugs. To enhance efficiency, we opted for an adaptive platform trial, entitled RECLAIM. Adaptive platform trials are efficient because effective and ineffective treatments can be identified in a timely fashion using repeatedly performed interim analyses with predefined stopping rules. Furthermore, additional promising treatments—often based on external evidence delivered by other studies—can be added to an existing protocol soon after they have been identified, and fewer controls (patients receiving a placebo or usual care) are needed [13].

The RECLAIM protocol is modular and consists of a core protocol, treatment-specific appendices (TSAs), and country-specific appendices (CSAs). The core protocol describes the procedures that apply to all trial domains and participating countries. The TSAs describe the details of each investigational product (IP) and its control, and IP-specific exclusion criteria, outcomes, and adaptations to the default trial assessments schedule. The CSAs describe the trial sites and usual PCC care in each participating country. RECLAIM currently only includes one participating country (the Netherlands), and this manuscript therefore focuses on the core protocol and the CSA for the Netherlands. Details about the various IPs that are being evaluated in RECLAIM will be described in future publications reporting the primary results for each specific IP. This protocol paper was written following SPIRIT guidance (see SPIRIT checklist in Appendix 1).

## Trial design

**Fig. 1:**
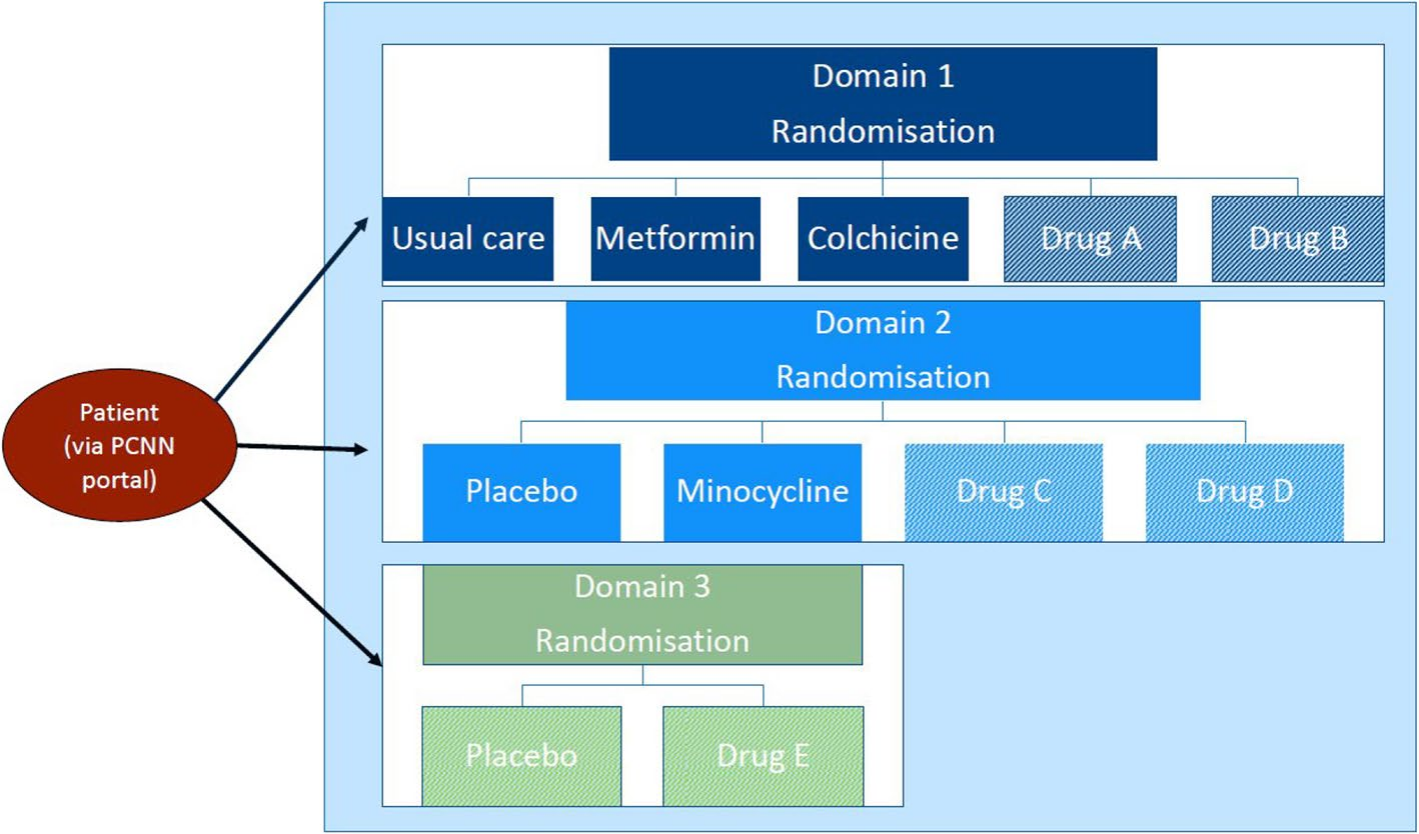
RECLAIM trial design. Abbreviations: PCNN, Post-COVID Network Netherlands. The RECLAIM trial consists of multiple domains, which are organised by the type of control group. The first domain, which was initiated in 2025, is an open-label domain comparing metformin and colchicine with a usual care control group. This domain could in the future be expanded with additional drugs, denoted as drug A and drug B. The second domain was initiated in 2026 and compares minocycline to a matching placebo. This domain could also potentially be expanded in the future, provided that the matching placebo also matches the drugs to be added (denoted as drug C and drug D). If that is not the case, a new domain would have to be initiated (denoted as drug E and placebo, where this placebo is a different placebo from the placebo in the second domain)

**Fig. 2:**
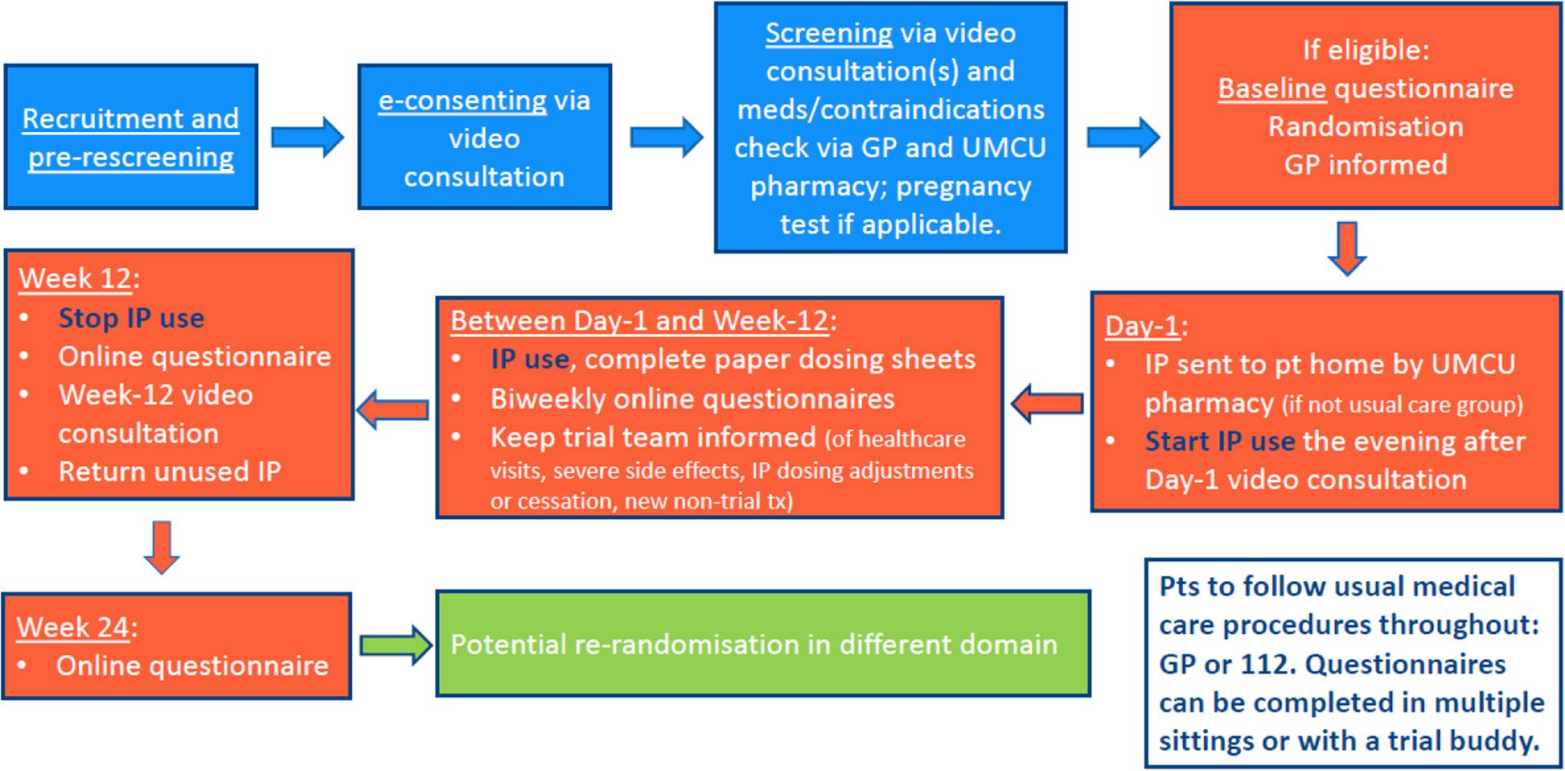
RECLAIM schedule of contact moments. Abbreviations: GP, general practitioner; IP, investigational product; UMCU, University Medical Center Utrecht. In the case of a placebo-controlled domain, placebo use will be initiated, monitored, and stopped in the same manner as IP use

RECLAIM is a phase III, randomised, controlled, adaptive platform trial. Randomisation occurs within trial domains. A trial domain consists of one or multiple IPs, each to be compared to one usual care arm, or one or multiple IPs, each to be compared to a matching placebo (Fig. 1). RECLAIM prefers placebo controls to minimise placebo effects, but a usual care control group may be chosen for financial and feasibility reasons. All repurposed drugs that are currently being evaluated in RECLAIM, or will be evaluated in the future, are widely available within Europe for another therapeutic indication than PCC. All study procedures are implemented remotely, using video consultations, eConsenting, trial product delivery to participants’ homes (if applicable), and online questionnaires (Fig. 2).

Within each trial domain, adult PCC patients can be enrolled if they are eligible for at least one IP arm and its control arm. An individual patient is randomised with equal probability to each arm for which s/he is eligible. When multiple domains are recruiting simultaneously, trial staff will assign participants to a domain based on practical considerations. If a patient is not eligible for the initial domain that s/he was screened for, but may be eligible for another domain that is also actively recruiting, s/he may be rescreened for that other domain. Participants are allowed to participate in only one trial domain at a time, but can be rerandomised into a different trial domain after completing the final follow-up assessments at week 24. Rerandomisation within a domain with multiple IP arms is not allowed.

## Objectives and outcomes

The primary objective of RECLAIM is to determine the impact of 12 weeks of IP use compared to the respective control group, on patient-reported health-related quality of life (HRQoL), using the Patient-Reported Outcomes Measurement Information System Profile29 (PROMIS-29) [14] physical health summary score as the primary outcome (Table 1). The framework is superiority. Secondary objectives are to determine the impact of 12 weeks of IP use on PROMIS-29 mental health summary scores and topic domain scores, and on specific PCC symptoms using other patient-reported outcome measures (PROMs) [15, 16]; to evaluate the safety and tolerability of each IP; and to evaluate the durability of IP treatment responses at week 24. The secondary outcomes are described in Table 1. The exploratory objectives and outcomes are also described in Table 1 and include all the above stratified by PCC disease phenotype and pre-enrolment PCC symptom duration. We consider it likely that IP efficacy differs by disease phenotype and duration, but a priori stratification is not yet possible because the underlying disease mechanisms are insufficiently understood.

**Table 1:** RECLAIM objectives and outcomes

Primary objective	Outcomes for the primary objective
1. To determine the impact of each IP vs. control on patient-reported HRQoL	Assessed at 12 weeks (end of trial product use period), adjusted for the baseline value:^a^ ● PROMIS-29 physical health summary score (ref 14)
Secondary objectives	Outcomes for the secondary objectives
2. To determine the impact of each IP vs. control on patient-reported mental HRQoL and on the seven PROMIS-29 domains individually	Assessed at 12 weeks, adjusted for the baseline value:^a^ ● PROMIS-29 mental health summary score (ref 14) ● PROMIS-29 domain scores (ref 14): physical function, fatigue, pain interference, depressive symptoms, anxiety, ability to participate in social roles and activities, sleep disturbance
3. To determine the impact of each IP vs. control on patient-reported specific PCC symptoms: ● Fatigue ● PEM ● Cognitive functioning ● Autonomic dysfunction	Assessed at 12 weeks, adjusted for the baseline value:^a^ ● CIS, fatigue severity subscale score (ref 15) ● DSQ-2 PEM score (ref 16) ● PROMIS cognitive function 8a score (ref 14) ● DSQ-2 POTS score (ref 16)
4. To determine safety of each IP in PCC patients	Assessed at 12 weeks: ● Frequency of related and unrelated (S)AEs and SUSARs in each IP arm including their severity and outcome (MedDRA-coded)
5. To determine tolerability of each treatment in PCC patients	Assessed at 12 weeks: ● Percent of doses missed for each IP and placebo (if available) ● Percent of participants with adequate adherence for the specific IP
6. To evaluate the durability of IP treatment responses	Assessed at 24 weeks (12 weeks after cessation of trial product use): For each of the scores in 1–3 above, the proportions of participants with treatment success at week 12 that maintain or exceed their week 12 score level at week 24
Exploratory objectives^b,c^	Outcomes for exploratory objectives^b,c^
7. Explore the above by PCC disease phenotype	● Same as above but stratified by phenotype
8. Explore the above by pre-enrolment PCC symptom(s) duration	● Same as above but stratified by pre-enrolment PCC symptom(s) duration
9. Investigate PCC pathophysiology and mechanisms of action of potential treatments	● Described in relevant TSAs
10. Identify biomarkers for PCC symptom clusters and treatment(s)-associated recovery	● Described in relevant TSAs

*Abbreviations:* *CIS* checklist individual strength (reference 15), *DSQ-2* DePaul Symptom Questionnaire version 2 (reference 16), *HRQoL* health-related quality of life, *PCC* post-COVID condition, *PEM* post-exertional malaise, *POTS* postural orthostatic tachycardia syndrome, *PROMIS-29* Patient-Reported Outcomes Measurement Information System Profile29 (reference 14), *SAE* serious adverse event, *SUSAR* suspected unexpected serious adverse reaction, *TSA* treatment-specific appendix

^a^The effect size will be summarised by the posterior mean and median of the estimated coefficients for each IP arm compared to the control arm, together with 95% credible intervals

^b^Additional outcomes may be described in relevant TSAs

^c^Performance-base, imaging, and/or biomarker outcomes may be added if funding permits

## Trial population and eligibility criteria

The trial population consists of adult, clinically stable PCC patients, residing in the Netherlands. Patients should have had persistent PCC symptoms, including fatigue and/or PEM, for a period of at least 12 weeks after the onset of a SARS-CoV-2 infection (see Table 2 for permitted diagnostic options). The symptoms should not have been present prior to this index infection, but may have partially subsided and resurged after the infection. The patient must agree to the exchange of relevant medical information between their healthcare providers and the RECLAIM team to support safety and efficacy evaluations. Several general and domain-specific exclusion criteria apply (Table 2). The domain-specific exclusion criteria concern contraindications to each specific IP, and these are described in detail in each respective TSA. Participants are allowed to continue chronic use of concomitant medications for PCC or for any other reason, provided that they are stable on those medications and they can be safely used in combination with trial medications (Table 2). RECLAIM follows the Heads of Medicines Agencies contraception and pregnancy testing in clinical trials recommendations for IPs that are contraindicated in pregnancy [17].

**Table 2:** RECLAIM eligibility criteria

**Inclusion criteria—for all**	**Exclusion criteria—for all**
Adults aged 18 years or older	Having been diagnosed with (exacerbation of) a chronic disease that can account for the onset of the PCC-like symptoms
Residing in the study area for the duration of trial participation^a^	Being hospitalised or institutionalised at screening. Patients can be rescreened after discharge Presence of a serious medical condition that would prevent completion of follow-up
Persistent PCC symptoms, including fatigue and/or PEM, for a period of at least 12 weeks after the onset of a SARS-CoV-2 infection. The symptoms were not present prior to the infection, but may have partially subsided and resurged after the infection	Currently enrolled, or having been enrolled within the last 30 days, in any other study with interventions or procedures that may affect RECLAIM outcomes or procedures. Individuals can be rescreened after at least 30 days have passed since participation in the other study has been completed
Self-reported confirmation of having had a SARS-CoV-2 infection by:^b^ a. Positive SARS-CoV-2 nucleic acid amplification test, such as PCR b. Positive SARS-CoV-2 rapid diagnostic test, including home-administered tests c. COVID-19 diagnosis by a treating physician (GP or medical specialist), based on the above or other clinical tests and assessments Alternatively, a treating physician diagnosed the patient with PCC, thereby implicitly acknowledging that the patient had COVID-19 in the past Will not be verified in medical records	Having initiated chronic use of a new licensed medication for any indication in the 3 months prior to the eligibility confirmation by a trial physician. This criterion may be reassessed after sufficient time has passed Willing to commit to not starting any chronic use of new licensed medications for any indication between eligibility confirmation and week 12 unless medically necessary as determined by a treating physician
Willing and able to provide informed consent	**Exclusion criteria—domain-specific**^c^
Willing and able to perform trial procedures	The participant cannot be randomised to at least one IP arm and its control arm within a trial domain due to (refer to relevant TSAs for details): - Known hypersensitivity to an active IP ingredient or IP/placebo excipient - Receiving a treatment that is contraindicated to a trial IP - Already using a trial IP, or a drug in the same drug class as a trial IP, outside of the trial - Any other reason why a trial IP cannot be used, such as (risk of) pregnancy or breastfeeding or renal insufficiency
Allowing their GP/treating physicians/pharmacy and the RECLAIM trial team to exchange medical information that is relevant for the participant’s safety and trial assessments	

*Abbreviations: GP* general practitioner, *IP* investigational product, *PCC* post-COVID condition, *PCR* polymerase chain reaction, *PEM* post-exertional malaise, *TSA* treatment-specific appendix

^a^Questionnaires currently only available in Dutch but additional languages may be made available and would be described in the respective country-specific appendix

^b^The trial will start using the SARS-CoV-2 infection definition described above. However, SARS-CoV-2 testing has declined significantly from 2023 onwards. We may therefore have to include probable SARS-CoV-2 infections in the future. A probable SARS-CoV-2 infection would be defined as having had a flu-like illness episode during a SARS-CoV-2 epidemic wave (as determined by wastewater and sentinel surveillance) that preceded post-infection chronic illness resembling PCC (including fatigue and/or PEM)

^c^These criteria will be assessed by licensed trial physicians. They will question the patient and verify prescription drug and contraceptive use, and other medical exclusion criteria (see TSAs), in each individual patient’s medical and/or pharmacy records. In case pregnancy is contraindicated, RECLAIM follows the procedures described in reference 17, which may include a pregnancy test. A renal function check may be required for specific drugs in specific populations (see TSAs). Additional IP-specific in- and exclusion criteria, if any, are listed in relevant TSAs. The TSAs also describe the latest information about drug interactions and contraindications as per the IP’s SmPC/PIL

## Sample size

Simulations of the Bayesian trial design for the primary outcome were performed to select the maximum sample size and to determine the type I error rate and power of the trial. In the simulations for the first domain, we assumed a normal distribution of the PROMISE-29 physical summary score with a mean of 45 and a standard deviation of 10, and we defined a clinically meaningful difference as at least 2.5 points improvement [18]. Interim analyses will take place after the first 100 participants in a specific IP arm completed follow-up and each 50 thereafter, with a maximum sample size of 500 per arm. This Bayesian approach with interim analyses will likely reduce the overall sample size required compared to a frequentist approach. The mean sample size at which a statistical conclusion is reached is estimated at 192 per trial arm, with 95% power and a one-sided type I error probability of 2.1%. The total expected sample size for the first domain is therefore about 576. More details are available in the statistical analysis plan for the first domain (Appendix 2). Additional statistical analysis plans will be developed for each new domain.

## Contact moments and assessments

Figure 2 shows a schedule of contact moments and Table 3 a schedule of assessments. RECLAIM uses the Your Research platform (Amsterdam, the Netherlands) to manage participant registration, planning of contact moments, and eConsenting, Castor Electronic Data Capture (Amsterdam, the Netherlands) for online data entry by RECLAIM staff and participants, and Microsoft PowerBI (Redmond, WA, USA) to visualise trial progress and flag concerns requiring follow-up by RECLAIM staff.

**Table 3:** Schedule of assessments

	All visits and assessments are conducted and monitored remotely	Pre-screening to day 1	Day 1	Weeks 2–10	Week 12	Week 24
Pre-screening	Via phone, with verbal consent	X				
Informed consent	Videocalls, incl. eConsenting	X				
Eligibility confirmation	Offline medical record verification and videocall. May include home pregnancy testing	X				
Randomisation		X				
Baseline questionnaire	Online questionnaire (by pt from home)	X				
IP prescription and final contraindications check by pharmacy		X				
IP/placebo dispensing	If randomised to IP/placebo	X				
Day 1 procedures	Videocall + online questionnaire		X			
IP/placebo use	If randomised to IP/placebo		Start	X	End	
Week 2 to week 10 procedures	Online questionnaires			X		
Week 12 procedures, incl. final IP/placebo accountability	Videocall + online questionnaire. Includes review of dosing diary and pill counts only if randomised to IP/placebo				X	
Week 24 procedures	Online questionnaire					X
Online questionnaire topics	Sociodemographics/lifestyle	X				
	Reproductive, general medical, COVID-19 and PCC history	X				
	Concomitant medications	X				
	Updates of all of the above			X	X	X
	Current PCC symptoms	X		X	X	X
	HRQoL (PROMIS-29 + CIS-fatigue)	X	X	X	X	X
	Other PROM questionnaires	X		X	X	X
	Safety, pregnancies		Start	X	End	[X]
	Trial product adherence			X	X	
	Trial product acceptability				X	
	Other assessments (refer to TSAs)	[X]		[X]	[X]	[X]

*Abbreviations:* *CIS* checklist individual strength (reference 15), *HRQoL* health-related quality of life, *PCC* post-COVID condition, *PROM* patient-reported outcome measurement, *PROMIS-29* Patient-Reported Outcomes Measurement Information System Profile29 (reference 14), *TSA* treatment-specific appendix

The RECLAIM team receives lists of potentially interested and eligible patients from the post-COVID Network Netherlands PCC patient portal team (http://www.postcovidonderzoek.nl/). Over 16,000 PCC patients have voluntarily registered in this portal. RECLAIM staff contact potential participants by phone to obtain verbal consent for pre-screening and discuss key eligibility criteria. If a patient appears potentially eligible, a licensed trial physician or nurse conducts a videocall to obtain formal voluntary informed consent via eConsenting and to initiate screening. The screening process involves multiple steps, including at least one videocall. Final eligibility is always determined by a trial physician. Most eligibility criteria are assessed through direct questioning of the patient, but all medical eligibility criteria are verified by asking the patient’s general practitioner to complete a paper-based checklist. If a pregnancy test is required, the patient receives a test kit by regular mail and is asked to present the result to a trial physician during a videocall. If the patient is still eligible up to this point, s/he is randomised to a trial arm within that domain.

The University Medical Center (UMC) Utrecht outpatient pharmacy is responsible for dispensing the IP and placebo (if applicable) following randomisation. Prior to dispensing, pharmacy staff apply their standard procedures to check for drug appropriateness (i.e. contraindications and drug-drug interactions). Any discrepancies between the drug appropriateness assessments conducted by the trial physicians and pharmacy staff are resolved through discussion. Randomised patients are withdrawn if a relevant contraindication or drug-drug interaction was missed prior to randomisation. Although randomising after the final pharmacy drug appropriateness check could potentially reduce such early withdrawals, this approach was considered impractical due to the significantly increased workload it would impose on the pharmacy.

Participants in placebo-controlled domains are blinded throughout their trial participation, whereas those in open-label domains are not. To minimise expectation bias, open-label participants are asked to complete the baseline questionnaire before being informed of their randomisation outcome. After completion of the baseline questionnaire, participants receive printed instructions at home about trial procedures in general and about their trial product if applicable. Those assigned to a trial product additionally receive the product as well as a printed dosing diary for daily intake recording. A day 1 videocall is conducted with all participants after they have received these materials to confirm readiness for trial initiation. Participants in a trial product arm are instructed to begin using the product in the evening of the day 1 videocall.

The default product use duration is 12 weeks. Participants randomised to a product are asked to complete online follow-up questionnaires on day 1, at 2, 4, 6, 8, and 10 weeks, at the end of the 12-week product use period (week 12), and 12 weeks after product cessation (week 24). Participants in a usual care arm follow the same schedule and complete identical questionnaires (see Table 3 for a list of questionnaire topics) except for items related to product accountability, adherence, and tolerability. A week 12 videocall is conducted with all participants to maximise day 1 to week 12 data completeness and to follow-up on any outstanding adverse events or other concerns. For those in a trial product arm, this videocall also includes final product accountability, including a review of the completed dosing diary and a count of any remaining product. Participants are then asked to take left-over product to their local pharmacy. At week 24, all participants receive a final request to complete an online questionnaire to assess the durability of treatment effects.

## Randomisation and blinding

Participants are randomised using a secure, fully validated, and regulatory-compliant online system. Randomisation is conducted within each trial domain using block randomisation with variable block sizes equal to 1, 2, or 3 times the number of arms in that trial domain. In domains with more than two arms, randomisation is stratified based on the specific arms for which each participant is eligible. For example, in the case of two IP arms A and B, and a usual care arm, three separate randomisation sequences are generated: one for participants eligible for A and usual care only, one for those eligible for B and usual care only, and one for those eligible for all three arms. Equal allocation probabilities are applied across all eligible trial arms.

Open-label domains are not blinded. Placebo-controlled domains are double-blinded: participants and RECLAIM staff who have direct contact with participants are blinded. The trial statistician is not blinded. This was not deemed necessary because he will follow a statistical analysis plan that was developed a priori (Appendix 1), and this way, he can interact with the data safety monitoring board (DSMB) directly. Unblinding is done at the level of individual participants. UMC Utrecht pharmacy staff with direct participant or trial staff contact are blinded, but a dedicated research team within the pharmacy is not blinded. They can implement unblinding procedures 24/7 when deemed necessary to properly assess the safety of an individual participant. The DSMB members receive unblinded interim analysis results from the trial statistician, but procedures are in place to ensure that these results (even in blinded format) are treated with strict confidentiality outside the DSMB to minimise the risk of inadvertently influencing trial investigators, staff, or participants.

## Risks, burdens, and safety reporting

RECLAIM will only assess IPs that are hypothesised to alleviate PCC symptoms and to be safe in PCC patients, based on the literature, clinician and patient experiences, and/or underlying disease mechanisms. These IPs must be approved for other indications. Though used off-label in the RECLAIM trial, IPs will never be used above their authorised dose.

The exclusion criteria were designed to reduce IP-associated risks as much as possible, and medical exclusion criteria are verified as described above. GPs of participants are informed of their patient’s RECLAIM participation and randomisation group or, if blinded, the unblinding procedures. They are also provided with the contact details of the trial team, including a 24/7 emergency phone number. Participants receive a wallet card with that same information to show to any healthcare professional that they consult with during their trial participation. Participants are instructed to contact the trial team in case of trial-related medical questions and to follow regular healthcare procedures in case of medical emergencies (whether or not trial-related). They are also instructed to inform the trial team if they have had a healthcare contact for any reason during their 12-week product use period.

RECLAIM participation may be burdensome to PCC patients because they suffer from severe fatigue, and some may not be mobile. To accommodate participants as much as possible, they are allowed to complete online questionnaires in multiple short sittings, and/or assign a trial buddy to assist them with trial procedures.

RECLAIM collects information about all side effects experienced by trial participants, whether they are expected (i.e. listed in the relevant Summary of Product Characteristics) or unexpected (not listed). RECLAIM procedures differentiate between two types of side effects: (1) side effects for which participants sought medical advice or made (temporary) dosing adjustments and (2) side effects for which they did not take any action. Participants complete questions about both types of side effects in the online questionnaires (systematic reporting) and are asked to report side effects of the first type to the trial team when they occur (participant-initiated reporting). Participants may also report side effects of the second type to the trial team spontaneously in between questionnaires, but this is not required, and trial staff do not encourage them to do so. Only side effects of the first type that occur in the 12-week product use period in trial product arms are considered adverse events (AEs). AEs are documented individually in dedicated Castor forms, coded using the Medical Dictionary for Regulatory Activities (MedDRA) [19], and included in the annual safety reporting. All serious adverse events (SAEs; using the standard definition) [20] and pregnancies in all trial arms that occur between day 1 and week 24 are also documented in dedicated Castor forms, MedDRA-coded, and included in the annual safety reporting. However, only pregnancies that occur in trial product groups during the 12-week product use period are followed up until after delivery, unless a longer duration is specified in the TSA (which is, for example, the case for colchicine due to its teratogenic properties). Suspected unexpected serious adverse reactions (SUSARs) can by definition only occur during IP use. They are reported in the EudraVigilance portal following EudraVigilance regulations [21]. A medical monitor supervises all safety assessment and reporting procedures, and safety reports are uploaded in European Clinical Trials Information System (CTIS) annually. We will report all harms in an aggregated MedDRA-coded manner in the publications dedicated to the primary outcomes.

## Adherence and discontinuation

To maximise retention and data completeness, participants are not considered lost to follow-up until the end of their 24-week participation period. They receive email requests to complete each online questionnaire and are asked to do so within 5 workdays after the online questionnaire is made available. They receive automated email reminders within those 5 workdays. After the 5-day window has passed, trial staff continue attempts to motivate or assist the participant to complete the questionnaire, but only for the baseline, day 1, week 12, and week 24 questionnaires. However, the trial team may only contact the participant a maximum of three times per missed questionnaire to minimise the perception of coercion. To maximise adherence to trial product use, participants using a product are asked about their adherence as part of the online questionnaires, using their dosing diary as a memory aide. The dosing diaries themselves are reviewed by the participant and a staff member together during the week 12 videocall. All participants are instructed to contact the trial team in case of difficulties with any type of protocol adherence to discuss the reasons of non-adherence.

Participants may withdraw from the trial at any time for any reason. The site principal investigator may withdraw a participant if there are concerns about that participant’s safety. Any individual IP arm, or the entire platform trial, may be temporarily suspended or prematurely terminated by the sponsor or the research ethics committee if there is sufficient reasonable cause, including concerns about participant safety, protocol compliance, or data quality. Individual IP arms may also be terminated if IP efficacy or futility, as defined below, has been demonstrated.

## Data analysis

A Bayesian analysis of covariance (ANCOVA) model with prior distributions is used for the primary analyses of domains consisting of multiple trial arms, while a frequentist approach may be used for the primary analyses of domains with only two arms and for secondary or exploratory analyses. The statistical analysis plan for the primary analyses of the first open-label domain using a Bayesian approach is included as Appendix 1. Interim analyses are conducted after the first 100 participants in a specific IP arm completed follow-up and each 50 thereafter. The primary analyses will be performed according to the intent-to-treat principle. Missing outcome values will be imputed using a last observation carried forward approach, with a sensitivity analysis using multiple imputation. Efficacy is to be concluded if the Bayesian posterior probability that the IP leads to an increase in the PROMIS-29 physical health summary score at follow-up exceeds 99.5%. Futility is to be concluded if the Bayesian posterior probability that the IP leads to an increase of at least 2.0 points in the PROMIS-29 physical health summary score at follow-up is below 5%. If an IP arm is declared efficacious or futile, the trial will continue with the remaining arms. This process will continue until a statistical conclusion for each IP is reached or a maximum of 500 participants per arm have completed trial participation. Results of the primary analysis will be presented using the posterior mean and the 95% credible interval of the treatment effect parameters.

## Ethics and dissemination

RECLAIM adheres to the ethical principles of the 75th World Medical Association Declaration of Helsinki (Helsinki, Finland, October 2024). The trial dossier was submitted for ethical approval via CTIS (EUCT number 2024–511580-28-02; trial documents, including the participant information sheets and informed consent forms, available at https://euclinicaltrials.eu/search-for-clinical-trials/?lang=en&EUCT=2024-511580-28-02), was approved by the NedMec Medical Ethics Committee in the Netherlands (dossier number D-25–500086), and was registered in ClinicalTrials.gov (identifier NCT07280572). Participants will not receive any monetary or other compensation for completing trial procedures. No provisions were made to provide participants with access to trial products after their trial participation has ended. RECLAIM participants are covered by the UMC Utrecht clinical trial insurance policy.

Primary trial results will be shared with participants and other PCC patients through online webinars and plain-language summaries distributed via email, as well as published on the websites and social media channels of the post-COVID Network Netherlands, UMC Utrecht, and the European Clinical Research Alliance on Infectious Diseases (Ecraid). Primary results will also be reported in the designated formats in CTIS and ClinicalTrials.gov. Academic dissemination of primary, secondary, and exploratory results will occur through publications in international peer-reviewed journals, inclusion in a PhD thesis, and presentations at national and international conferences. Additionally, clinically relevant findings will be proactively communicated to members of clinical guideline committees to support evidence-based updates. We will follow the authorship guidelines issued by the International Committee of Medical Journal Editors (ICMJE) [22]. Final authorship decisions will be made by the RECLAIM sponsor PI (JHHMvdW).

## Trial governance and administration

The RECLAIM sponsor is the UMC Utrecht, in Utrecht, the Netherlands. The only participating country thus far is the Netherlands. RECLAIM is being implemented fully remotely by one trial site based at Ecraid, in Utrecht. The first two domains of the trial were funded by the Netherlands Organisation for Health Research and Development (*ZonMw* in Dutch) and the Long COVID Foundation (*Stichting Long COVID* in Dutch). The trial protocol and other documents were developed by the sponsor and Ecraid; the funders did not contribute and did not have any authority over the content. RECLAIM is a core component of the Post-COVID Network Netherlands, which is a collaboration of more than 30 Dutch institutes and organisations including PCC patient organisations (https://postcovidnet.nl/). The public is not involved in RECLAIM design or conduct.

RECLAIM is governed by a trial steering committee (TSC), which includes two PCC patients, and a DSMB (see acknowledgments for all names and affiliations of TSC and DSMB members). The DSMB reviews the interim analysis results and formulates recommendations to the TSC; the TSC decides which actions to be taken. These processes are described in detail in the TSC and DSMB charters, which can be accessed in the CTIS trial dossier (https://euclinicaltrials.eu/search-for-clinical-trials/?lang=en&EUCT=2024-511580-28-02).

Data management is done by a data management team at the UMC Utrecht, and trial conduct monitoring by a clinical research associate at Ecraid who is not part of the data collection team, in accordance with data and privacy management and monitoring plans.

## Discussion

Clinical guidelines are based on robust evidence, most reliably obtained through randomised controlled trials. Traditionally, this requires conducting multiple trials that each examine a limited set of interventions for a specific condition. While this approach remains the benchmark for generating clinical knowledge, it is inherently resource-intensive, time-consuming, and lacks adaptability when new insights emerge during or outside the trial process. The recent COVID-19 pandemic demonstrated the value of adaptive platform trials, such as the REMAP-CAP trial, as a practical alternative [13]. Although numerous treatment approaches for PCC have been proposed, and many studies are underway, the availability of results from randomised controlled trials remains limited. Meanwhile, emerging research continues to generate hypotheses about potentially effective interventions. The aim of the RECLAIM platform trial is to address this gap by strengthening the evidence base for PCC clinical management and, in doing so, to improve care for a large global patient population.

## Trial status

This manuscript outlines the RECLAIM core protocol version 5.0, dated 7 August 2025, and the CSA for the Netherlands version 4.0, dated 5 January 2025. The procedures described have been implemented since the activation of the trial site on 17 February 2025. Only minor modifications have been made since site activation, except for the addition of the minocycline TSA in May 2025, which received approval on 4 September 2025. Recruitment for the first domain (metformin, colchicine, and usual care) commenced on 18 February 2025 and the first interim analysis is planned for February 2026. Recruitment for the second domain (minocycline versus placebo) began on 3 February 2026. We cannot estimate a date on which recruitment will be completed due to the adaptive nature of the trial. It depends on the outcome of the interim analyses for each individual IP. The trial is designed to continue evaluating additional IPs until one of the following conditions is met: (1) PCC is no longer considered a public health concern; (2) the efficacy of all plausible IPs has been established and no further candidates remain; or (3) funding is no longer available.

## Supplementary Information

Additional file 1: SPIRIT checklist

Additional file 2: Statistical analysis plan

## Data Availability

JHHMvdW, JvR, JMHS, ERL, and JSS will have access to the final datasets. Third parties may request access to the data for use in other PCC-related research via DataverseNL (https://dataverse.nl/dataverse/UMCU). All requests will be reviewed by at least three members of the RECLAIM TSC, and approved or rejected (with explanation) by the RECLAIM Sponsor PI (JHHMvdW). Unrestricted open access to the complete trial product-specific datasets is not possible because not all participants have consented to share their data with all types of third parties. They provided separate permissions for commercial and/or non-commercial entities, in or outside the European Economic Area. Moreover, it is essential to ensure that the main aim of the RECLAIM trial—facilitating the incorporation of evidence-based effective treatments into clinical guidelines—is protected.

## Abbreviations

AE: Adverse event
COVID-19: Coronavirus disease 2019
CSA: Country-specific appendix
DSQ: DePaul Symptom Questionnaire
DSMB: Data Safety Monitoring Board
Ecraid: European Clinical Research Alliance on Infectious Diseases
EU CTIS: European Clinical Trials Information Systems
GP: General practitioner
HRQoL: Health-related quality of life
IP: Investigational product
PCC: Post-COVID condition
PEM: Post-exertion malaise
POTS: Postural orthostatic tachycardia syndrome
PROM: Patient-reported outcome measurement
PROMIS-29: Patient-Reported Outcome Measurement Information System Profile29
SAE: Serious adverse event
SARS-CoV-2: Severe acute respiratory syndrome coronavirus 2
SUSAR: Suspected unexpected serious adverse reaction
TSA: Treatment-specific appendix
TSC: Trial steering committee
UMC: University Medical Center
