# Supplementary material for: The RECLAIM adaptive platform trial for the evaluation of treatments for post-COVID condition in the Netherlands: core protocol
Source: Trials. 2026 Feb 19;27:238. doi: 10.1186/s13063-026-09570-1 (PMC13020272; doi:10.1186/s13063-026-09570-1)
Supplement: Supplementary file 2 — Additional file 2: Statistical analysis plan [file 13063_2026_9570_MOESM2_ESM.pdf]

RECLAIM: Adaptive Platform Trial for the Evaluation of Treatments for Post-Acute Sequelae of SARS-CoV-2 Infection (PASC)
Statistical Analysis Plan

**Short title**	RECLAIM Statistical Analysis Plan
**EU trial number**	2024-511580-28-00
**Sponsor (in Dutch: verrichter/opdrachtgever)**	University Medical Center Utrecht, Netherlands
**Principal investigator (in Dutch: hoofdonderzoeker/uitvoerder)**	Janneke van de Wijgert, MD PhD MPH<br>Julius Center for Health Sciences and Primary Care<br>University Medical Center Utrecht,<br>Utrecht University, Utrecht, the Netherlands
**Funding party**	The initial trial is funded by the Dutch governmental funder ZonMw and foundation Stichting Long Covid.

DOCUMENT HISTORY

Document	Date of version	Summary of Changes
**Statistical analysis plan version no.**	*<Insert date>*	*<Only include the most important changes>*
**Version 1**	January 27, 2025	Not applicable
**Version 2**	September 30, 2025	Revised criteria for adequate adherence

CONFIDENTIALITY STATEMENT

This document contains confidential information that must not be disclosed to anyone other than the sponsor, the investigative team, regulatory authorities, and members of the Research Ethics Committee.

TABLE OF CONTENTS

1. Summary ... 5

2. STATISTICAL ANALYSIS ... 5

2.1 Analysis sets ... 5

2.2 Randomisation and blinding ... 6

2.3 Sample size, trial power and level of significance used ... 6

2.4 Statistical analyses ... 7

2.4.1 Participant demographics and other baseline characteristics ... 7

2.4.2 Primary efficacy analysis ... 7

2.4.3 Analysis secondary endpoint(s) ... 8

2.4.4 Analysis other study parameters/endpoints ... 9

2.5 Procedure for accounting for missing, unused and spurious data ... 10

2.6 (Statistical) criteria for termination of the trial ... 10

2.7 Procedure for reporting any deviation(s) from the original statistical plan ... 10

ABBREVIATIONS

AE	Adverse Event
ANCOVA	Analysis of Covariance
COVID-19	Coronavirus Disease 2019
CrI	Credible Interval
DSMB	Data Safety Monitoring Board
ECRAID	European Clinical Research Alliance on Infectious Diseases
EU/EEA	European Union/European Economic Area
HRQoL	Health-related Quality of Life
IP	Investigational Product
ITT	Intention To Treat
LOCF	Last Observation Carried Forward
PASC	Post-Acute Sequelae of SARS-CoV-2 Infection
PI	Principal Investigator
PROM	Patient-Reported Outcome Measurement
PROMIS	Patient-Reported Outcome Measurement Information System
SAE	Serious Adverse Event
SARS-CoV-2	Severe Acute Respiratory Syndrome Coronavirus 2
SAP	Statistical Analysis Plan
SOP	Standard Operating Procedure
UMCU	University Medical Center Utrecht

1. SUMMARY

This document presents the Statistical Analysis Plan (SAP) for the usual-care domain of the RECLAIM trial (EU trial number: 2024-511580-28-00). The RECLAIM trial is a multi-arm, randomised, controlled, adaptive platform trial, with as main aim to identify efficacious and safe treatments (with a focus on repurposed drugs or devices) for patients with post-acute sequelae of SARS-CoV-2 infection (PASC). The current version of this document only discusses the SAPs for the usual care (open-label) domain, though SAPs for other domains may be added in the future. The statistical analysis of the usual-care domain uses Bayesian statistics for the primary and secondary efficacy analyses. Some secondary and all exploratory analyses use frequentist statistical analysis methods. The study procedures are detailed in the study protocol.

2. STATISTICAL ANALYSIS

2.1 Analysis sets

The primary efficacy analysis sets will be created for each IP separately. The final primary efficacy analysis set is used for the primary analysis at the completion of the open-label domain. The interim primary efficacy analysis sets are used for the interim analyses. The final primary efficacy analysis set for a specific IP will consist of the participants who meet all of the following conditions:

- The participant is eligible to receive the IP in question.
- The participant was randomized to either the IP in question or the open-label control, at a time when the IP arm was open for recruitment.
- The participant completed the PROMIS-29 physical health score at baseline and at least once at weeks 2, 4, 6, 8, 10 or 12.

The interim primary efficacy analysis sets for a specific IP will consist of the participants who meet all of the following conditions:

- The participant is eligible to receive the IP in question.
- The participant was randomized to either the IP in question or the open-label control, at a time when the IP arm was open for recruitment.
- The participant completed the PROMIS-29 physical health score at baseline and at least once at weeks 2, 4, 6, 8, 10 or 12.
- The participant started participation in the trial (i.e. had a day 1 video call) at least 14 weeks before the database lock date of the interim analyis.

The definition of the final and interim primary efficacy analysis sets follows the Intention To Treat (ITT) principle.

The secondary efficacy analysis sets will be created for each IP separately. The secondary efficacy analysis set for a specific IP will consist of the participants who meet both of the following conditions:

- The participant is eligible to receive the IP in question.
- The participant was randomized to either the IP in question or the open-label control, at a time when the IP arm was open for recruitment.

The definition of the secondary efficacy analysis sets follows the Intention To Treat (ITT) principle, but participants who did not complete the relevant outcomes at baseline and at least once during follow-up will be excluded as appropriate.

Two per protocol analysis sets will be defined for the final efficacy analysis:

- A per protocol analysis for participants with adequate adherence to the trial medication. The definition of this per protocol analysis set follows the definition of the final primary efficacy analysis set, with the additional requirement that participants in an IP group had at least 80% adherence to the IP, based on the review of dosing diaries at 12 weeks. Adequate adherence is defined as the participant using at least 198 pills of metformin in the metformin group (where 247 pills would be expected given the protocol) or 134 pills of colchicine in the colchicine group (where 167 would be expected given the protocol) during the first 12 weeks of follow-up. For the care-as-usual group, this per protocol analysis is equivalent to the final primary efficacy analysis set.
- A per protocol analysis for all participants with completed outcome data at 12 weeks. The definition of this per protocol analysis set follows the definition of the final primary efficacy analysis set, with the additional requirement that participants have filled out the PROMIS-29 at 12 weeks.

For each IP, a safety analysis set will be defined as all participants who were randomized to receive the IP in question.

2.2 Randomisation and blinding

Participants will be randomised using a secure, fully validated, and compliant web-based randomisation system. Randomisation will be done by trial domain, using block randomisation with variable block size of 1, 2 or 3 times the number of arms within that trial domain. In the case of 3 arms, the variable block sizes will be 3, 6 and 9. Randomisation will be stratified for the trial arms that the participant is eligible for. In the case of two IP-arms A and B, and a usual care arm, three separate randomisation sequences will be generated: for those eligible for A only, those eligible for B only, and those eligible for A and B. We will use equal allocation probabilities per trial arm. In case of 3 arms, the allocation ratio will be 1:1:1, such that 33% of participants are randomised to each arm. In case of 4 arms, this will be 1:1:1:1 and 25%. The number of IP arms may vary over time as new IPs are added and others are terminated. Participants can only be randomised within one trial domain at a time (based on which trial domains are active, and if multiple domains are active, the patients' own choice), but can be rerandomised in a different domain after completing 24-weeks in their original domain. Rerandomisation within the same usual care domain with multiple treatment arms will not be allowed but may be reconsidered as the trial evolves. The trial clinician conducting the randomisation will only be informed about the treatment allocation of the individual patient. Overall accrual in each treatment arm, as well as the balance between arms, will be monitored centrally.

The initial usual care domain will be open-label, with only data managers and analysts blinded for allocation. Results of interim analyses will be shared with the DSMB members together with the unblinding key, but care will be taken to keep even the blinded results of interim analyses confidential as much as possible to avoid influencing trial investigators, staff or participants.

2.3 Sample size, trial power and level of significance used

For the usual care domain, simulations of the Bayesian trial design for the primary endpoint were performed to determine the trial's frequentist operating characteristics (type I error rate and power).

These simulations were also used to calculate the sample size at which a statistical conclusion of efficacy or futility of the primary endpoint is expected to be reached. The frequentist operating characteristics were performed with 10,000 randomly generated data sets. The analysis model in these simulations was a Bayesian analysis of covariance (ANCOVA) model with model formula and prior distributions as specified in Section 2.4.2 below. The type I error rate was calculated under the null hypothesis of no treatment effect of the IP. Power was calculated as the (frequentist) probability of declaring an IP efficacious given an assumed treatment effect parameter of 2.5 points (on the PROMIS-29 physical health summary score) for that treatment. For the simulations, we assumed that the presence of a second IP, with a slightly lower treatment effect parameter of 2.0 points.

Based on the simulation results, the maximum sample size was set to 500 patients per arm. The simulations showed that the mean and median number of participants to be included in each trial arm are 192 and 150, respectively, and that the adaptive design has 94.8% power and a one-sided type I error probability of 2.1%. In addition to the trial design features already described, the simulations incorporated the following assumptions:

- Analyses will be performed according to the intent-to-treat principle.
- Sequential analyses will be done after the first 100 participants per IP arm have accrued, and then after every 50 additional participants per IP-arm.
- Efficacy is to be concluded if the Bayesian posterior probability that the IP leads to an increase in the PROMIS-29 physical health summary score at follow-up exceeds 99.5%.
- Futility is to be concluded if the Bayesian posterior probability that the IP leads to an increase of at least 2.0 points in the PROMIS-29 physical health summary score at follow-up is below 5%.
- If an IP arm is declared efficacious or futile, the trial will continue with the remaining arms.
- This process will continue until a statistical conclusion for each IP is reached or a maximum of 500 participants per arm have completed trial participation.

2.4 Statistical analyses

2.4.1 Participant demographics and other baseline characteristics

Demographic and other baseline characteristics will be described using descriptive statistics (means and standard deviations for normally distributed outcomes, medians and interquartile ranges for ordinal and skewed continuous outcomes, and frequencies and proportions for categorical outcomes). A separate baseline table will be made for each IP and its control arm, based on the secondary efficacy analysis sets. No statistical tests will be used to compare the demographic and other baseline characteristics between the randomisation groups among all included participants, because the trial design guarantees that both groups are drawn from the same population. However, in the primary and secondary efficacy analysis sets, patients who have not reported any outcomes after baseline are excluded, which might lead to differences in patient characteristics. For the primary and secondary efficacy analysis sets, the distribution of the PROMIS-29 and relevant patient characteristics at baseline will be compared between randomisation groups using independent samples t-tests, Mann-Whitney or chi-square tests, as appropriate.

2.4.2 Primary efficacy analysis

In the usual care domain, the primary efficacy analyses consist of interim primary efficacy analyses and the final primary efficacy analysis. The interim primary efficacy analyses will be performed after 100, 150, 200, 250, 300, 350, 400 and 450 patients have been randomized per arm and have had 14 weeks of follow-up time, using the interim primary efficacy analysis sets. The final primary efficacy analyses will be performed a) after 500 patients have been randomized per arm and have had 14 weeks of follow-up time, or b) the IP's treatment arm is terminated, whatever comes earlier. The final primary efficacy analyses will be performed using the final primary efficacy analysis sets.

The statistical model for the primary efficacy analysis will consist of an ANCOVA model for the PROMIS-29 physical health summary score at 12 weeks, with adjustment for trial arm and the baseline PROMIS-29 physical health summary score. This model will be analysed using a Bayesian statistical framework, and the effect of each IP will be analyzed in a separate model. In each sequential analysis, posterior probabilities for efficacy and futility are calculated separately for each IP arm compared to its control arm. The effect size will be summarised by the posterior mean and median of the estimated coefficients for the IP arms compared to the usual care control arm, together with 95% credible intervals (95% CrI's) of these coefficients. The analysis of the primary endpoint uses the same assumptions and prior distributions as in the sample size calculation described in Section 2.3.

For each IP, the model equation of the ANCOVA model is given by:

$$Promis - 29\ at\ 12\ weeks = \beta_0 + \beta_1 IP + \beta_2 \text{Promis-29 at baseline} + \epsilon,$$

where the error term ϵ is assumed to have a normal distribution with mean 0 and variance σ^2. *IP* is a binary variable indicating whether a patient has been randomized to the IP or to control. Participants who received another IP are not included in the model. β_1 describes the treatment effect of the IP. A normal-inverse-gamma prior is assumed for the model parameters β_0, β_1, β_2 and σ^2.The scale and shape parameters of the inverse-gamma prior for σ^2 (the variance of the outcome) are set to 0.1. A multivariate normal distribution with mean vector μ_0 and variance-covariance matrix $\frac{\sigma^2}{\kappa_0} I_3$ (where I_3 represents the identity matrix with 3 rows and 3 columns) will be used as prior for the coefficients β_0, β_1 and β_2, conditional on the variance σ^2. κ_0 will be set to 0.001 and μ_0 will be set to the vector [45,0,0].

The normal-inverse-gamma prior for β_0, β_1, β_2 and σ^2 is a conjugate prior, which means that the posterior distribution of these parameters is also a normal-inverse-gamma distribution. To obtain posterior results, the posterior distribution is obtained in closed form, and posterior summaries are based on 1 million samples from the posterior.

2.4.3 Analysis secondary endpoint(s)

In secondary analyses, Bayesian ANCOVA models will be used to analyze the treatment effects on the following outcomes:

- the PROMIS-29 physical health summary score at 24 weeks after randomisation
- the PROMIS-29 mental health summary score at 12 and 24 weeks
- the PROMIS-29 domain scores for physical function, fatigue, pain interference, depressive symptoms, anxiety, ability to participate in social roles and activities, and sleep disturbance at 12 and 24 weeks
- the Checklist Individual Strength (CIS-8R) score at 12 weeks
- the score on the DePaul Symptom Questionnaire (DSQ-2) PEM questions at 12 weeks
- the PROMIS cognitive function 8a score at 12 weeks
- the score on the DSQ-2 POTS questions at 12 weeks

These ANCOVA models will use the same priors and other settings as are used for the primary efficacy analysis, except that the first element of the mean vector μ_0 will be set to the population mean of the relevant outcome. The analysis of the scores at 24 weeks after randomisation serve to assess the durability of the intervention effect. These analyses will be performed in the secondary efficacy analysis population.

In another analysis, interaction between the treatment effect of the IP and disease phenotype as well as illness duration will be assessed. Disease phenotype will be defined as a categorical variable, and the interaction of this categorical variable with the IP variable will be added to the ANCOVA model. Illness duration will also be defined as a categorical variable based on the date of the initial Covid-19 infection (e.g. initial infection before or after March 2021). The interaction of this categorical variable with the IP variable will also be added to the ANCOVA model of the primary efficacy analysis. If these analyses provide evidence for interaction (i.e. effect modification), the interaction analyses will be repeated for the domain scores and the mental health summary scores of PROMIS-29 at both 12 and 24 after randomisation.

To evaluate the trajectory of the treatment effects during follow-up, linear mixed models will be developed for the primary outcome. The outcomes in the linear mixed models will be the physical and mental health summary scores of the PROMIS-29, based on the available measurements at baseline, 2, 4, 6 ,8, 10, 12 and 24 weeks after randomisation. The independent variables will be time since baseline (coded as a categorical variable), randomisation arm and their interaction. Within-subject correlations will be modeled using one of the following three approaches: a) a random intercept (at the participant level), b) a random intercept and a random effect of time since baseline, c) an unstructured covariance matrix of the repeated measurements at the different timepoints (baseline and 2, 4, 6 ,8, 10, 12 and 24 weeks after randomisation). The Bayesian information criterion will be used to choose among these three model specifications. In separate analyses, relevant demographic and clinical characteristics will be added as independent variables. The linear mixed models will be estimated with a frequentist approach, using restricted maximum likelihood estimation. The results will be presented as the estimated treatment effect of an IP for each time point separately, based on the difference in estimated marginal means and its 95% Confidence Interval (CI).

The results of the linear mixed models for the physical health summary score of the PROMIS-29 are also used to assess possible bias in the primary efficacy analysis due to imputation of missing values using the last observation carried forward approach. The estimated treatment effect after 12 weeks in the linear mixed model will therefore be compared with the results of the primary efficacy analysis.

Adverse events (AEs) and Serious Adverse Events (SAEs) will be tabulated and summarized by organ type for the safety analysis sets using line listing. No formal statistical tests are planned for the analysis of (S)AEs. The number of pregnancies during follow-up will also be reported for each randomisation group.

The level of adherence to the treatments will be assessed in each IP group using descriptive statistics, based on information in the dosing diaries.

2.4.4 Analysis other study parameters/endpoints

Not applicable.

2.5 Procedure for accounting for missing, unused and spurious data

In the primary efficacy analyses, missing values of the PROMIS-29 physical health summary score at 12 weeks will be imputed using the available measurements of this outcome at weeks 2, 4, 6, 8 and 10 using a Last Observation Carried Forward (LOCF) approach. The LOCF approach could lead to bias in the estimated treatment effects, for example if the strength of the treatment effects changes during the 12 week follow-up period and if the amount of missing observations differs by randomisation arm. In sensitivity analyses, the robustness of the estimated treatment effects to the missing data approach will be assessed. Two alternative methods are considered:

- Estimation of the treatment effect based on a linear mixed model, as described in Section 2.4.3 above. The linear mixed model automatically accounts for missing data in the outcome, based on the assumption that the missing data are missing at random.
- Multiple imputation using a Bayesian approach will be used to generate imputed values of the PROMIS-29 outcome at 12 weeks and to estimate the parameters of the Bayesian ANCOVA model, using the JointAI package in R. In this analysis the priors of the primary efficacy analysis will be adopted.

Multiple imputation with fully conditional specification will be used to impute missing data of relevant baseline characteristics in the linear mixed model analyses.

2.6 (Statistical) criteria for termination of the trial

The criteria for terminating an individual treatment arm are described in Section 2.3, and criteria for discontinuing the entire trial in Section 11.1 of the study protocol.

2.7 Procedure for reporting any deviation(s) from the original statistical plan

Post-hoc deviations of the statistical analysis plan will be annotated as such in trial reports and manuscripts. A motivation for the deviation will be provided in a manuscript supplement or appendix.
